# Research progress and limitation analysis of RNA interference in *Haemonchus contortus* in China

**DOI:** 10.3389/fvets.2023.1079676

**Published:** 2023-02-24

**Authors:** Bin Hou, Ying Hai, Buhe Buyin, Surong Hasi

**Affiliations:** ^1^Key Laboratory of Clinical Diagnosis and Treatment Technology in Animal Diseases, Ministry of Agriculture, College of Veterinary Medicine, Inner Mongolia Agricultural University, Hohhot, China; ^2^Wushen Animal Disease Prevention and Control Center, Ordos, China

**Keywords:** RNA interference, *Haemonchus contortus*, identification of gene function, limitation analysis, research progress

## Abstract

*Haemonchus contortus* is a highly pathogenic and economically important parasitic nematode that affects small ruminants worldwide. While omics studies hold great promise, there are fewer research tools available for analyzing subsequent gene function studies. RNA interference (RNAi) technology offers a solution to this problem, as it especially allows for the knockout or shutting off of the expression of specific genes. As a result, RNAi technology has been widely used to explore gene function and disease treatment research. In this study, we reviewed the latest advancements in RNAi research on *Haemonchus contortus* in China, with the aim of providing a reference for the identification of key genes involved in growth and development, anthelmintic resistance, diagnostic markers, and diagnostic drug targets for the treatment of *Haemonchus contortus*.

## 1. Introduction

*Haemonchus contortus (H. contortus)* is a type of parasitic nematode that belongs to the *Haemonchus* spp. (Trichostrongylidae) and is a major threat to the economically important gastrointestinal nematode of small ruminants. It primarily infects the abomasum, and when 2,000 or more worms are present there they can cause severe harm. These worms can suck up to 30 mL of blood per day, in addition to the blood loss caused by their departure. This can result in severe infection in livestock and cause many issues, such as anemia, weight loss, and even death (1). It is believed that *H. contortus* lays more eggs than other nematodes of the Trichostrongylidae family, with females laying 5,000–10,000 eggs every day. Third-stage larvae (L3) are more resistant and can generally survive for 3 months in pastures, and they can remain dormant for up to 1 year in adverse environments, leading to the contamination of pastures and the environment. Recent epidemiological studies showed that the positivity rate of *H. contortus* infection in sheep in Iran, India, Egypt, Brazil, and Ethiopia was 84.6, 44.7, 38.8, 76.4, and 68.75%, respectively (2–6). These developing countries have experienced significant losses in their dominant animal product industries due to *H. contortus* infections.

The prevention and treatment of *H. contortus* can be accomplished through the use of anthelmintics, with the selection and breeding of livestock breeds that are resistant to parasitic infection, biological control methods, and improved feed management (7, 8). Among these methods, anthelmintics are the most effective and economical means of control. Since the invention of highly effective and broad-spectrum anthelmintics, their prolonged use has led to serious resistance problems. Researchers have made extensive efforts to address these problems (9–11). With the release of the complete genome of *H. contortus* in 2013 (chromosome-level assembly was completed in 2019) (12) and the advancement of post-genomics in the last 15 years (13, 14), there are numerous molecular targets that can be exploited for control. Although these molecular targets have the potential to control *H. contortus*, they still require the evaluation of some techniques before they can be used as drug screening or vaccine candidates (15).

**Figure 1:**
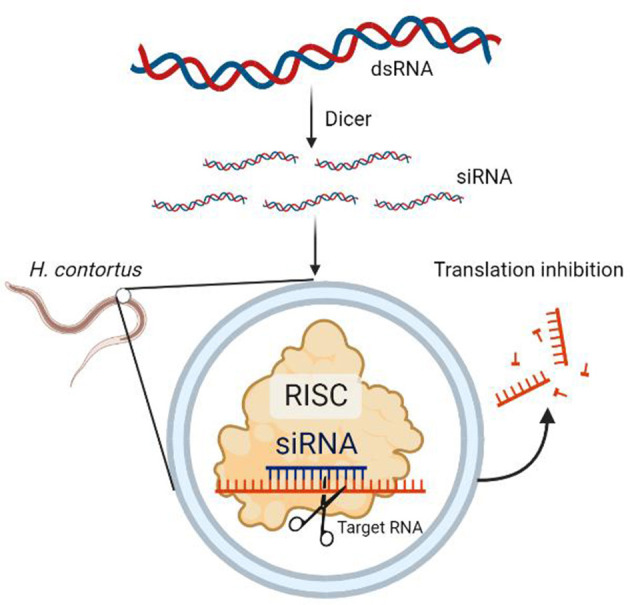
RNAi principle.

RNA interference (RNAi) is an important tool for evaluating the gene function. It is based on the principle that exogenous genes, such as viral genes, artificially transferred genes, transposons, and so on, are randomly integrated into the host cell genome and often produce some double-stranded RNAs (dsRNAs), which are transcribed using the host cell. Dicer, a nucleic acid endonuclease in the host cell, cleaves the dsRNA into multiple small fragments of RNA, commonly known as small interfering RNA (siRNA), with a specific length and structure. These siRNA fragments are unwound into a righteous strand and an antisense strand by intracellular RNA decapping enzymes and are then bound by antisense siRNA to some enzymes *in vivo*. The RNA-induced silencing complex (RISC) specifically binds to the homologous region of exogenous mRNA and functions as a nuclease, cleaving the mRNA at the binding site, which is the area where the antisense strand of the siRNA is complementary to it. The cleaved and fragmented mRNA is then degraded, leading to a degradation response in the host cell against these mRNAs (Figure 1).

In recent years, the availability of genomic and transcriptomic data for *H. contortus* has highlighted the need for functional genomics techniques. Since there are limited methods to directly study gene function in nematodes, RNAi and its related technologies have been developed over time to successfully identify key genes, provide a rational method to develop new control strategies, and apply them in relevant gene function studies (16). Comparative genomic analyses, especially using data from free-living *Caenorhabditis elegans* (*C. elegans*), can help predict gene function (17). In this study, we reviewed recent studies on the RNAi technique applied to *H. contortus* development genes and drug target screening and discussed the problems and limitations of the technique. It provides a theoretical basis for further in-depth research on the application of RNAi technology in parasitic nematodes.

## 2. Research progress of RNAi for *Haemonchus contortus*

### 2.1. Key genes for growth and development

**Figure 2:**
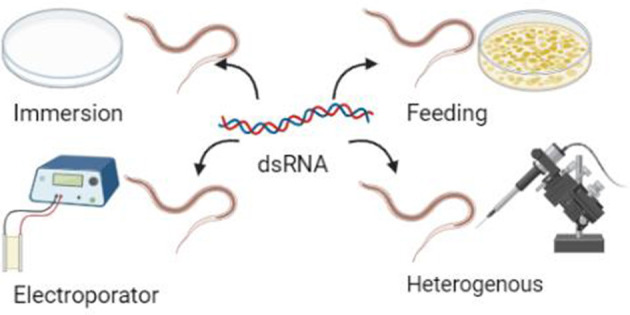
Four different methods of dsRNA introduction.

The exploration of the free-living and parasitic stages of *H. contortus* has never ceased. Free-living third-stage larvae (L3) of *H. contortus* can tolerate extreme weather conditions such as desiccation (18). To explore the molecular mechanism of desiccation survival, researchers screened two desiccation-related differentially expressed genes, *Hc-ubq* and *Hc-gst*, in L3 using RNA-seq (19). Quantitative RT-PCR results showed that expression was upregulated in L3 in desiccation environments, and silencing both *Hc-ubq* and *Hc-gst* reduced the survival rate of L3. Silencing of the homologs of *C. elegans, Ce-ubq-2*, and *Ce-gst-7* also showed higher larval mortality, which suggests that *ubq* and *gst* may play an important role in nematode dehydration tolerance. It is reported that researchers significantly reduced the transcript abundance of two transcript isoforms of the serine/threonine-specific protein kinase, *AKT, Hc-akt-1a*, and *Hc-akt-1b*, by using the soaking RNAi method (20). The results showed that silencing these two genes significantly reduced *in vitro* larval stage development. In addition, research shows that silencing of the *Hc-tgfbr2* genes significantly reduced the development of L3–L4 *in vitro* (21, 22). These genes play an important role in regulating development and the transition from free-living to parasitic stages (Figure 2).

The silencing of genes for motility at different stages of *H. contortus* was also reported by Andrew C Kotze, who found that *in vitro* dsRNA soaking of two β-microtubulin genes, *tub8-9* and *tub12-16*, restricted motility in L3, but adults did not exhibit the phenomenon (23, 24). This may be because phenotypic disruption is caused by multiple factors, including the transient nature of gene repression and the variability of RNAi action at different developmental stages. The different stages of RNAi also simultaneously demonstrate that dsRNA uptake is non-specific and that L3 can silence specific genes without normal mouthparts. Effective silencing of the *Dim-1* gene in *H. contortus* L3 resulted in reduced migration of L3 and slowed the larval development from L3 to early L4.

*Matrix metalloprotease 12A* (*MMP-12*) is known to have important roles during embryonic development, organ morphogenesis, and pathological processes in animals. Recent studies used three different siRNAs targeting the *Hc-MMP-12* gene for silencing. Compared to the snRNA-treated control, siRNA-2 silencing of the *Hc-MMP-12* gene resulted in a shorter *H. contortus* length (25). This was in addition to a 54.02% egg reduction, a 16.84% reduction in the hatching rate, and a 51.47% reduction in infection intensity in the treated group compared to the control. A new gene, *Hc-clec-160*, was screened by RT-PCR. To characterize the function of the *Hc-clec-160* gene, earlier studies on the interference of *clec-160* in *C. elegans* using heterogeneous RNAi resulted in shortened body length and reduced egg size, indicating that *clec-160* plays an important role in the growth and reproduction of this helminth (26). Using the same approach, a new gene called the *Hc-fau* gene was also shown to play an important role in regulating the life cycle and reproduction in *H. contortus* (27).

The dauer of *H. contortus* is similar to that of *C. elegans* and *hookworm in vivo* (28). Growth and development cease at a precise point in the L3; metabolism levels are low, and seasonal variations are evident. Protection of the sheath and the dauer are the main survival strategies in the free-living and parasitic stages in the absence of the host or adverse conditions. The discovery of the steroid ligands of *DAF* opened a new avenue of small molecule physiology in *C. elegans*. These signaling pathways are present in parasitic nematodes and have been indicated to play a role in development regulation and the formation of L3 during the infectious process. *Hc-daf* is similar to *ce-daf* in affecting worm development and competence. This study demonstrated that *daf-3, daf-5, daf-12, daf-16* and *daf-22* inhibited the development of L3 into L4 after RNAi (29–34). If we can obtain some clues as to the genes or signal transduction mechanisms related to the dauer and prevent its stagnation in the host, we will solve the fundamental occurrence of spring *H. contortus* outbreaks (Table 1).

**Table 1:** Reported methods for the study of gene function using RNAi in parasitic nematodes.

**Target gene**	**Parasite species and Stage and delivery method**	**Delivery method**	**RNAi effect**	**References**
*Hc-ubq, Hc-gst*	*H. contortus* (L3)	Feeding	Transcript levels were reduced by 66.4 and 44.2%	(19)
*Hc-akt-1a, Hc-akt-1b*	*H. contortus* (xL3)	Soaking	Transcript levels were reduced by 68.8 and 63.0%	(20)
*Hc-tgfbr2*	*H. contortus* (xL3)	Soaking	Fewer xL3 developed to L4	(21, 22)
*tub8-9, tub12-16*	*H. contortus* (xL3, L4, and adult)	Soaking	Treated L3 worms were less able to migrate through a filter mesh, indicating decreased motility, and showed less development to the L4 stage	(23, 24)
*Hc-MMP12*	*H. contortus* (L3)	Soaking	Reduced the number of eggs (54.02%), hatchability (16.84%), and worm burden (51.47%)	(25)
*Hc-clec-160*	*C. elegans* (adult)	Heterogeneous, feeding	Confers an extended lifespan, increased lipid storage in the intestine, and shortened body length	(26, 27)
*Hc-daf-22*	*C. elegans* (L1)	Feeding	Egg numbers of Hc-daf-22 RNAi adults decreased, larvae grew slower, and the average life span was shorter than that of N2 wild type	(29)
*Hc-daf-3*	*H. contortus* (xL3)	Soaking	Retarded L4 development	(30)
*Hc-daf-5*	*H. contortus* (xL3)	Soaking	Retarded xL3 development	(31)

### 2.2. Screening of anthelmintic resistance genes and anthelmintic targets

The problem of anthelmintic resistance in *H. contortus* is a serious threat to parasitic control, and in some areas, anthelmintics such as macrolides and benzimidazoles have completely lost their deworming effect on gastrointestinal nematodes in livestock. The identification of alternative anthelmintic targets is an increasingly important task. Using RNAi for the functional identification of relevant genes and the search for new compounds that display antagonistic activity against these genes to enhance anthelmintic efficacy opens new prospects (35). An RNAi-based functional validation assay revealed *HCON_00143950* as an IVM (ivermectin)-tolerance-associated gene in *H. contortus*. The possible role of this gene in IVM resistance could be the detoxification of xenobiotics in phase I of xenobiotic metabolism (36). In France, researchers performed an RNAi of the *nhr-8* gene in an ivermectin-resistant strain of *H. contortus* (37). The *nhr-8* silencing increased the susceptibility of the resistant strain to ivermectin and mediated the upregulation of genes associated with ivermectin detoxification. It is also reported that silencing the *Cyt-P450* gene resulted in reduced resistance to ivermectin, which led to larval pharyngeal paralysis and thus starvation (36). Meanwhile, silencing the *Cyt-P450* gene increased the sensitivity of *H. contortus* to ivermectin; these results also provide new approaches for further understanding ivermectin targets.

Earlier studies have demonstrated that normal movement can be restored in *H. contortus* by using RNAi on *HcGlu Cl-*α*3* through the use of *C. elegans* as a model organism (38). Their results supported the claim that ivermectin has a paralyzing effect on parasitic nematodes by stimulating channels in the nervous motor system. Cholinergic agonists, such as levamisole and pyrantel, are widely used to treat nematode infections as anthelmintics. These drugs cause spastic paralysis in nematode parasites by activating acetylcholine receptors (AChRs) present in their body wall muscles. The critical role of *acr-8 in vivo* anthelmintic sensitivity is substantiated by the successful demonstration of RNAi gene silencing in *Hc-acr-8*, which reduced the sensitivity of *H. contortus* larvae to levamisole. Pyrantel sensitivity remained unchanged, thus providing new evidence for distinct modes of action of these important anthelmintics in parasitic species vs. *C. elegans* (39). As one of the largest protein families, they regulate nearly all processes within the cell and are considered important drug targets. Numerous studies have been conducted on inhibitors for protein kinases (protein kinases, *PKs*), leading to a wealth of compounds that target *PKs*, which have the potential to be lead anthelmintics. Some researchers used RNAi to identify 35 *PK* compounds against *C. elegans* and *H. contortus*. Of these, 18 compounds showed efficacy against *C. elegans*, and six other compounds also showed efficacy against at least one parasitic species (35). RNAi has great potential as a screening tool for identifying potential anthelmintic targets.

## 3. Analysis of the limitations of RNAi in the study on *Haemonchus contortus*

RNAi has been widely used in the functional identification genes of *H. contortus*. However, its problems are also evident and constantly arise in the following three respects.

First, the sensitivity of *H. contortus* to RNAi varies with dsRNA delivery methods and target genes and is less reproducible. The results of many studies have shown that commonly used immersion, feeding, and injection methods can produce RNAi phenomena in *H. contortus*, but the immersion method is less efficient and more costly, and the storage time of larvae and batch effects have a significant impact on the results (40). The heterogeneous RNAi makes it difficult to accurately deliver dsRNA to specific sites and can easily lead to cellular damage. The feeding method is still the most widely used and is more likely to produce phenotypic effects and be easy to observe (41). When dsRNA or siRNA is delivered using electroporation, the phenotypic effects cannot be examined due to the high mortality of the treated larvae (42, 43). RNAi may inhibit gene expression under certain conditions. However, this only works on a limited number of genes, and in some cases, the effect is negligible, which may reflect differences in dsRNA uptake and transport to and from cells in *H. contortus*. The efficiency of gene interference in *H. contortus* has no particularly clear correlation with the gene itself or the delivery method; however, existing research found that gene interference may only occur in certain cell types. For example, the *C. elegans rde-4* null mutants are amenable to RNAi with siRNA but not dsRNA (44). If parasitic nematodes lack *rde-4* or a functional equivalent, RNAi should still be possible with siRNA delivery. They can still be delivered by siRNA; that is, siRNA is more effective than dsRNA. It is very likely that the ortholog of *rde-4* or some undefined mechanism is still in operation. These examples can show that the efficiency of gene interference will become more controllable with further information and a more detailed search of genome sequencing projects. The insights gained from studies on other worms may also help optimize dsRNA delivery and determine which developmental stages may be best targeted.

Second, a single developmental stage for RNAi will make it difficult to assess phenotypes that may only be expressed at later developmental stages, as reliable culture systems have not been established. Further research is needed to develop reliable phenotyping systems. *In vitro*, reagents such as hypochlorite are widely used for sterilization and unsheathing of L3, which can affect larvae and subsequent stages of development even at very low concentrations (45). High concentrations of dsRNA delivery may be cytotoxic, so it may be necessary for us to determine the optimal concentration for each gene tested. It has also been reported that some of the phenotypes generated by RNAi can be replicated by chemical inhibitor target activity, providing proof of the principle that RNAi can replicate chemo-suppressive effects and confirming that RNAi screening is a potentially powerful and effective method for identifying novel drug targets. Based on RNAi, we need to further investigate the mechanism of these genes and improve the culture system of *H. contortus* to detect the long-term effects of RNAi; only then can RNAi become a reliable method for gene function detection.

In addition, there is also a large body of data indicating limitations in identifying potential *H. contortus* genes as a predictive model based on *C. elegans* data, with RNAi-mediated silencing being reliable for some genes but not others. *H. contortus*-specific genes are mainly located on autosomes, which have a higher recombination rate, and evolutionary mutations mainly occur in these regions, possibly being promoted by higher recombination. When these genes are silenced by RNAi, only a few genes can alter the phenotype, and in *C. elegans*, it is more difficult for us to observe the occurrence and alteration of these phenotypes. When conditions allow, we can further perform protein analyses to examine the decrease in mRNA and protein levels. Therefore, we need to be extra cautious and careful while using *C. elegans* as a model organism (46, 47). The RNAi technology will be more specific and targeted if these challenges are successfully overcome.

## 4. Discussion and perspective

The current database has a large amount of genomic and transcriptomic data, and many studies have identified certain important genes that can successfully eliminate *H. contortus*. We currently need to move forward to the next level; RNAi technology has significantly improved its efficacy and ease of use and can be used to develop a more reliable and functional genomics platform. This can also be combined with technologies such as CRISPR/Cas and will provide a more rational approach to gene function identification. *In vivo* experiments have currently demonstrated that site-specific targeting of genes can improve their success rate, making *in vivo* RNAi a feasible option for identifying essential gene functions during infection, which is particularly important for elucidating non-conserved genes and speculating on the genes that play a role in influencing host immune responses. However, greater efforts are still needed to improve our understanding of *H. contortus* biology and to identify new approaches for parasitic control and prevention. The conclusion that can be drawn from the outlined studies is that RNAi is possible in parasitic nematodes and, if effectively utilized, can be a valuable tool for screening and validating new gene and vaccine targets.

## Author contributions

SH: conceptualization. BH: writing—original draft preparation and writing—review and editing. YH: visualization. YH, BB, and SH: supervision, project administration, and funding acquisition. All authors read and agreed to the published version of the manuscript.
